# Design for success: Identifying a process for transitioning to an intensive online course delivery model in health professions education

**DOI:** 10.1080/10872981.2017.1415617

**Published:** 2017-12-25

**Authors:** Paige L. McDonald, Kenneth J. Harwood, Joan T. Butler, Karen S. Schlumpf, Carson W. Eschmann, Daniela Drago

**Affiliations:** ^a^ Department of Clinical Research and Leadership, George Washington University School of Medicine and Health Sciences, Washington, DC, USA

**Keywords:** Intensive courses, accelerated courses, curriculum design, online learning, e-learning

## Abstract

Intensive courses (ICs), or accelerated courses, are gaining popularity in medical and health professions education, particularly as programs adopt e-learning models to negotiate challenges of flexibility, space, cost, and time. In 2014, the Department of Clinical Research and Leadership (CRL) at the George Washington University School of Medicine and Health Sciences began the process of transitioning two online 15-week graduate programs to an IC model. Within a year, a third program also transitioned to this model. A literature review yielded little guidance on the process of transitioning from 15-week, traditional models of delivery to IC models, particularly in online learning environments. Correspondingly, this paper describes the process by which CRL transitioned three online graduate programs to an IC model and details best practices for course design and facilitation resulting from our iterative redesign process. Finally, we present lessons-learned for the benefit of other medical and health professionsʼ programs contemplating similar transitions.

**Abbreviations:** CRL: Department of Clinical Research and Leadership; HSCI: Health Sciences; IC: Intensive course; PD: Program director; QM: Quality Matters

## Introduction

Increasingly, medical and health professions educational programs are adopting e-learning as a supplement to existing face-to-face curricula or as a stand-alone course delivery model [1–3]. E-learning is a broad term indicating the incorporation of internet technologies within a wide variety of course models [4], from web-enhanced, to blended, to fully online courses. Benefits of e-learning include cost effectiveness and increased accessibility, flexibility, interactivity, self-direction, and self-efficacy in learning [5–8]. According to the Institute of Medicine (IOM), adoption of e-learning models is critical to actualizing the ‘Learning Health System’ of the future [9,10]. While existing literature offers advice on supplementing existing coursework with internet technologies [11], creating fully online healthcare-related courses for senior medical students [1], and evaluating the online portion of a blended pharmacist continuing medical education course [3], little guidance is actually provided on designing a fully online program of study in medical or health professions education. Yet, these professions can benefit from educational scholarship on the design of distance education.

**Figure 1.**
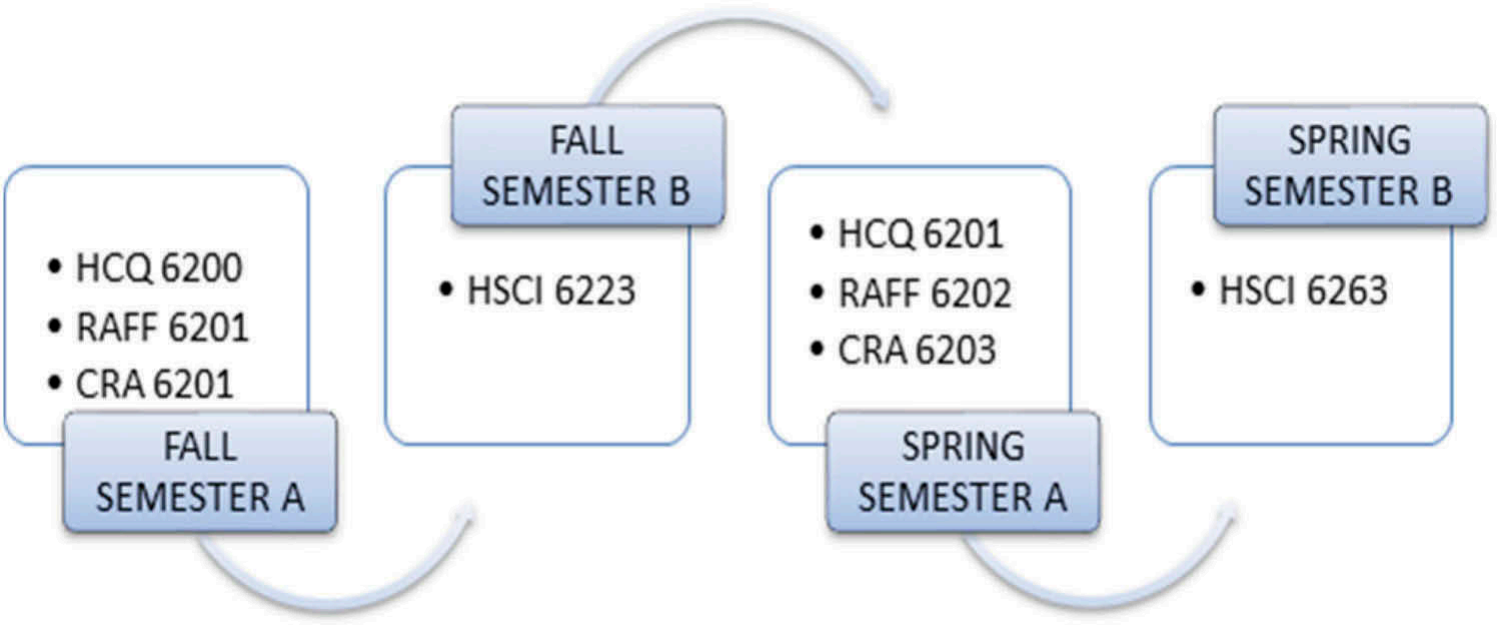
Example of initial course sequence for 7-week intensive curricula.

Distance education in the USA can trace its roots to the correspondence courses of the early 1900s [12]. Distance learning has been defined as a mode of delivery in which teachers and learners are separated and greater responsibility for learning is placed upon the learner [13]. E-learning is a more recent form of distance education facilitated through internet technologies. In 2014, Allen and Seaman reported the percentage of students taking online courses at 26% in 2013, an ‘all time high’ [14, p.4]. In addition, 90% of surveyed academic leaders indicated it 'likely' or 'very likely' within 5 years of the report that the majority of all higher education students will have taken at least one online course [14, p.5]. Moreover, 74% of the surveyed academic leaders rated the quality of online education as ‘the same as or superior to those as in face-to-face instruction’ [14, p.4]. Perhaps this response reflects the attention given to the quality of design and delivery of online education over the past few decades. In 2000, anticipating the need for quality guidelines, the Institute for Higher Education Policy published guidelines for the design and delivery of online learning, in which they argued that learning outcomes, not the existence of available technology, should drive course design and that facilitation must emphasize student-faculty interaction complemented by timely, constructive feedback.

More recently, non-profit, quality assurance organizations, such as Quality Matters (QM) (https://www.qualitymatters.org) have arisen to address the need for training in the design and facilitation of e-learning. The QM rubric provides an evidence-based approach to course design. The rubric, which is updated every three years based upon scholarly research, is in its fifth edition and comprises a set of 43 specific standards used to evaluate the design of online and blended courses based upon a pedagogical approach. QM also provides online training for faculty and instructional designers which emphasizes alignment between learning objectives, assessment, materials, learner activities, and course technology as critical to achieving course outcomes and designing an effective online or blended learning experience.

Intensive courses (ICs), or accelerated courses, are gaining increasing popularity in higher education [15–17]. ICs are defined as traditional (semester or quarter equivalent) courses presented in an accelerated (time shortened) manner which may involve fewer contact hours than traditional courses held over a fifteen or sixteen week time period [16,18–20]. As increasing numbers of non-traditional, working adult students enroll in higher education, institutions have adopted ICs to meet the needs of these learners who find it challenging to adhere to the traditional course delivery system (course length and delivery modality) adopted by most institutions [6,15]. Non-traditional students enrolled in ICs ‘tend to be slightly older and working students’ [17, p.1109]. ICs align with the needs of these working adult learners, particularly in postgraduate education [16].

Students prefer ICs for a variety of reasons. They are convenient [15]; they facilitate the efficient use of student time [21], and they allow for degree completion in a reduced amount of time [22]. Additional benefits include students remaining motivated throughout the course and being less frequently absent compared to that seen in traditional courses [21]. Moreover, both faculty and students think ICs promote a 'continuous learning experience' which supports students in connecting and synthesizing material, in large part due to their ability to focus on fewer classes at one time [15, para.13]. However, there are also disadvantages to IC participation. Students have less time to review material and complete assignments; thus, ICs require greater focus in a shorter timeframe [15,23]. Perhaps for this reason, some students find ICs challenging and more stressful than traditional length courses [15,16,23,24].

ICs have been adopted in medical and health professions education [25,26]. Sonnadara and colleagues found the IC model ‘highly effective at teaching and developing targeted surgical skills in first-year orthopaedic residents’ [26, p.745]. In fact, these authors recommend adoption of an intensive ‘bootcamp’ model at the beginning of residency to develop requisite technical skills [26, p.748]. IC models have been successfully used in other pre-residency and final year medical school courses, including musculoskeletal injury management in primary care [27], colonoscopy training [28], and surgical specialty skills preceding surgical residency [29]. With the advent of accelerated medical school education [30], an IC may be a viable method in streamlining traditional courses throughout medical school education.

Principle details adoption of an IC model for a health policy management course in an accelerated executive MBA program, noting 'insufficient guidance' in the literature on 'how to redesign traditional courses' [25, p.79] to support an educational theory guided approach to course redesign. Thus, little guidance exists for how to transition an existing traditional program into an intensive model of delivery.

While ICs may be gaining in popularity, research regarding their comparative effectiveness to traditional full semester courses is inconclusive and none of the existing comparative research considers online or blended models of learning delivery. Some findings suggest equivalent results in academic achievement and instructor ratings [18,24]. Kucsera and Zimmaro report no significant difference in instructor ratings; in fact, ICs received ‘slightly higher overall course ratings on student evaluations than did traditional courses’ [18, p.62]. Hall et al. [17] report that most studies indicate greater student success in ICs as compared to traditional courses; the remaining studies demonstrate equivalent success. Conversely, Whillier and Lystad conclude that students taught in a traditional mode cohort achieved “significantly higher final grades compared to the intensive mode cohort” [31, p.286]. Regarding test scores, some evidence suggests comparable results [15] or slightly higher results for ICs than traditional courses [32,33]. However, Petrowsky [34] found students in ICs performed worse on comprehensive examinations. Results related to student satisfaction or attitudes toward ICs are also mixed. Wlodkowski et al. [35] report that students’ overall attitudes toward ICs were positive in comparison to traditional courses; Whillier et al. [31] note equivalent findings regarding student satisfaction; whereas, Mishra et al. [23] find most students unhappy with ICs, noting mostly negative perceptions.

Although inconclusive, research comparing ICs to traditional length courses does offer insight into important design considerations. Effective course design, which focuses on learning objectives and supports the student’s achievement with effective materials, learning activities, and teaching strategies, is required for successful ICs [15,16,35]. When designing ICs, faculty must carefully consider the intensity of the workload and the time students have to review and learn course materials and complete assignments when selecting the types and quantity of materials that will support achievement of learning objectives [16]. Also, the learning cycle within an IC should support active engagement with learning content and provide opportunities for faculty and peer feedback and guidance [36].

While providing insight into course design requirements for face-to-face ICs, existing research does not suggest a process by which faculty can convert a traditional program of study to an IC model of delivery. Moreover, most of the above recommendations relate to the design of ICs that will be delivered in a face-to-face environment, not in an online delivery model. As e-learning gains increasing popularity in medical and health professions education and faculty seek to maximize the benefits of e-learning in intensive online offerings, additional guidance must be provided on how to shorten course length while ensuring comparable levels of learning and student satisfaction in fully online programs of study. Correspondingly, the purpose of this manuscript is to (1) describe our processes of transitioning three online graduate health professions programs from a traditional to an intensive format, (2) to detail best practices for course design and facilitation developed during our iterative redesign process, and (3) to discuss lessons-learned. We hope that this article will be helpful to other medical and health professions academic programs contemplating similar transitions.

## Our context

The Department of Clinical Research and Leadership (CRL) is housed within the George Washington University School of Medicine and Health Sciences whose mission is committed to teaching with creativity and dedication, healing with quality and compassion, and discovering with imagination and innovation ( https://smhs.gwu.edu/about/mission-vision ). During our process of transitioning to ICs, CRL consisted of seven undergraduate, 12 graduate, and one doctoral program delivered primarily in an online, asynchronous format that graduated approximately 150 students per year.

In spring 2014, two CRL graduate programs, Health Care Quality (HCQ) and Regulatory Affairs (RAFF), were selected to transition from the traditional fifteen-week course structure (15-week) to a seven-week, intensive format (7-week). This transition was a response to input from outside educational consultants who suggested that our student population, which consists primarily of working adult learners, prefers more efficient learning experiences consisting of shortened semesters with more concentrated, sequential courses. In spring 2015, the Clinical Research Administration (CRA) graduate program was the next program selected to make the transition.

The Senior Associate Dean of Health Sciences convened preliminary meetings in spring 2014 with representatives from the initial two programs and appropriate stakeholders (administration, student advising, etc.) to discuss the impending changes, to assess the effect on other programs of study, and to develop transition plans and budgets. This group formed our initial ‘steering committee’ for the project. He also convened meetings with other university partners to discuss the transition and the possible implications to their programs. Once the steering committee finalized the preliminary plans, the program directors (PDs) were tasked with developing individual processes to transition each program of study. The following description highlights the commonalities and differences each PD used to make the transitions.

## Our process

The following paragraphs detail our process for transitioning the 15-week curriculum into 7-week ICs. While iterative, in general we followed a progression of activities from updating program student outcomes and key competencies to evaluating and revising courses prior to and following initial offerings.

### Updating program student outcomes and key competencies

When faced with the task of transitioning entire programs from 15-week to 7-week courses, PDs began by evaluating current program student outcomes. While program student outcomes existed for most programs, they had not been recently updated. Therefore, the PDs believed it prudent to start by comparing existing outcomes to external resources related to program competencies, identifying gaps and redundancies in existing curricula, and developing new curricular plans to address any issues. This step allowed PDs to align program student outcomes with the key competencies required to achieve those outcomes.

All programs began by evaluating existing program student outcomes; however, the process for completion varied slightly among programs. If a PD had sufficient content knowledge of the curriculum and of the knowledge requirements of current practice, typically gained through a literature review or existing accreditation standards, he or she completed the review and identified the gaps and redundancies. If, on the other hand, the field of study was evolving or the PD required additional expertise, this task was accomplished by a team that included the PD and appropriate subject matter experts (SMEs), either internal, such as faculty teaching within the program, or external to the organization, such as an advisory board. PDs also found it helpful to review existing literature within their respective fields of study to assist with this step. This step yielded changes in required program student outcomes across all programs, but the degree of required changes varied across programs. The Health Sciences Curriculum Committee reviewed and approved all programmatic changes by program of study.

### Mapping the curricula

Having updated and/or approved existing program student outcomes, PDs began the process of mapping the revised program outcomes and/or competencies to existing courses to identify gaps and redundancies in the curriculum and determine whether the existing courses were adequate and appropriately sequenced to achieve desired outcomes. Curriculum mapping is a process by which faculty create a visual representation of the way in which curriculum is delivered within a program of study [37]. Originally developed for K-12 education, this process has become a valuable tool for promoting achievement of program competencies or goals in higher education and for promoting common understanding among faculty tasked with evaluating an existing program of study [38]. For this process, the PDs either collaborated with SMEs or utilized core faculty within their programs of study.

For our purposes, curriculum mapping was required at both the program and course levels. First, PDs and SMEs began by creating maps of the existing curricula in comparison to the revised program competencies. For this process, PDs created a matrix with program student outcomes and key competencies related to each outcome on the vertical axis and course objectives on the horizontal axis in the order that courses were currently offered within each program of study. Next, faculty identified where in the existing curricula outcomes were being introduced (I), developed (D), or mastered (M). Table 1 shows an example of one student learning outcome measure from the Health Care Quality program curricula. This process allowed our faculty to identify gaps or redundancies in current course offerings, which could later facilitate revision of specific courses. It also allowed them to determine the need to create new courses or eliminate existing courses to achieve the revised program student outcomes. Finally, curriculum mapping allowed the PDs to consider the requirements for specific sequencing of courses within each program of study. Prior to this project, our programs of study did not consistently emphasize a specific course sequencing beyond the requirement to take the first and last course in a given program at the requisite time. However, mapping the I, D, and Ms of existing courses, allowed recognition of the importance of appropriately sequencing courses to scaffold knowledge across the curricula and to allow multiple opportunities for outcomes development, particularly given the task of transitioning to a 7-week delivery model in which sufficient time for I, D, or M might not be permitted in any one course. After determining the appropriate course sequencing of their core courses and electives, program directors could then create a revised curriculum map.

**Table 1.** Example of curricula mapping.

Course Title	Student Learning Outcome V I. Distinguish quality improvement, patient safety, leadership, organizational, and research theories/standards applicable to healthcare
HCQ Course A: Introduction to Health Care Quality	**I**
HSCI Course A: Biostatistics for Clinical and Translational Research	**I**
HCQ Course B: Building a Quality Culture	**D**
HSCI Course B: Epidemiology for Clinical and Translational Research	**I**
HCQ Course C: The Health Care Quality Landscape	**D**
HSCI Course C: Topics in Health Care Leadership	**I**
HCQ Course D: Quality Improvement Science in Health Care	**D**
HSCI Course D: Health Care Enterprise	**D**
HCQ Course E: Health Care Quality Measurement, Data Management and Analysis	**D**
HCQ Course F: Patient Safety Systems	**D**
HCQ Course G: Leadership and Change in Health Care Quality	**M**

I: introduced, D: developed, M: mastered

The newly created curriculum maps provided tools by which faculty could evaluate requisite changes in existing courses. PDs followed a similar process to complete this step. They evaluated course objectives with regard to their ability to support achievement of the requisite level (e.g., I, D, or M) indicated in the curriculum map. If needed, they revised course objectives or created new objectives. This course map could then be used as a guide for any course revision from a 15-week to a 7-week delivery model.

In addition to core program courses, we have several graduate courses offered across multiple programs of study, which we refer to as the Health Sciences (HSCI) core curricula. These courses support development of cross-disciplinary outcomes related to leadership, issues and trends influencing healthcare, healthcare administration and strategic planning, biostatistics and epidemiology, and leading change within healthcare organizations. Once PDs for HCQ and RAFF individually completed the process of creating new curriculum maps, the PD for the HSCI core curricula worked with them to create a curriculum map of the HSCI courses that supports the achievement of identified competencies across the two programs of study. In addition, scaffolding the concepts within the discipline specific and the HSCI courses, allowed for the PD to delimit extraneous course content among the HSCI and program-specific courses and to more efficiently lead students to achievement of higher level competencies within Bloom’s taxonomy. So in any given semester, the program would offer a core programmatic course in the first 7-week session (the ‘A’ session) and offer a HSCI course in the second 7-week session (the ‘B’ session). The HSCI offerings would scaffold critical concepts across the successive B sessions. Figure 1 presents an example of course sequencing for all 7-week curricula.

When we compared the curriculum maps for the two initial programs, we recognized that the HSCI courses were offered at differing times across the initial two programs, which made it difficult to determine how to sequence the courses to promote the required I, D, or M of cross-disciplinary competencies to achieve program student outcomes. Consequently, we decided on a lock-step sequence of HSCI courses across all programs of study that would support the development of the cross-disciplinary competencies. The third program, CRA, benefited from this prior work, by adopting the lock-step sequence.

### Building new courses and identifying initial best practice guidelines

The process of creating new courses in each program of study evolved over time as our original steering committee engaged in regular meetings throughout the process of creating and evaluating initial courses. The first courses offered in each program of study served as our test cases, which informed the development of subsequent courses. Lessons-learned, which we detail later in this paper, related to amount and choice of readings, student workload, types, and sequencing of assignments.

Due to the distinct content within each program or study, no one overarching theoretical framework was applied in course redesign. Rather, we applied principles from QM to optimize course design and streamline course content while ensuring that all readings, materials, and assignments aligned to promote mastery of course objectives. All PDs had previously attended QM training. QM is an evidence-based approach to course design which involves applying a rubric to ensure a pedagogical approach to course design or redesign (https://www.qualitymatters.org). The concept of *alignment* serves as a major part of the QM rubric. Alignment occurs

‘when each of the critical components of a course (learning objectives, assessment, materials, learner activities, and course technology) work together to ensure that students achieve the desired learning outcomes. When a course is aligned, each of the components directly supports the learning objectives and anything extraneous to the objectives is avoided’ [39].

Applying QM guidelines while ‘condensing’ the course length often required difficult decisions regarding which materials and activities were ‘essential’ to promote content mastery and which were ‘supplementary.’ Through these discussions, we eliminated redundancies and streamlined curricula by only utilizing the essential material required to support achievement of course learning objectives. The delimited material was not essential to the alignment of the course. Rather, it was material provided as optional or as reinforcement of existing themes or basic concepts, which QM recommends avoiding [39]. Also, the sequencing and scaffolding of key concepts across courses within a specific program allowed for removal of redundant material across the curricula.

The alignment process also required reconsidering or restructuring assignments to allow for both assessment of competency attainment and efficiency in providing feedback critical to future assignments. At times, achieving both accuracy and efficiency in an assessment necessitated adopting new assignment types (e.g., narrated presentations rather than formal writing assignments) as formative and summative assessments.

The process of designing the new courses also facilitated the development of ‘guidelines’ for course development to ensure consistency in format and workload across courses within a program of study. This consistency in course design allowed students to focus less on course structure and more on course content while progressing through the accelerated program. As we progressed from developing the initial courses in our programs, utilizing feedback received from student course evaluations about course structure and content, we developed the following best practice guidelines for 7-week course design:

#### Course format

- Use a consistent structure for the online look and feel across courses in a program of study
- Ensure consistency of menu items across courses
- Adopt a similar structure to organize information in weekly folders

#### Course content

Ensure alignment between readings, activities, and assignments to course objective(s)

- Ensure adequate readings/materials to support achievement of course objectives but avoid ‘supplemental’ materials that are not essential
- Ensure major assignments allow for assessment of progress toward mastery of course objectives
- When possible, include only two or three major assignments within any given 7-week course (to allow maximum time for assignment evaluation and feedback to students)
- When possible, scaffold assignments to allow students to maximize their work to achieve mastery of objectives while receiving faculty feedback as the course progresses

#### Assignment timing and type

Require the first major summative assignment by Week 3 in any given course to allow for students to determine their progress prior to the last day to add/drop a course.

- Reconsider the types of final assignments included (e.g., can students demonstrate mastery of objectives through a PowerPoint presentation rather than a paper, which would allow for feedback from both faculty and peers on the assignment and, perhaps, save the faculty time in grading assignments?)
- Require the last major assignment by Week 6 in a 7-week course to allow for grading and administrative processing of students’ final grading
- Limit discussion questions in weeks where major assignments are due when possible

In addition to developing best practice guidelines for course structure, content, and assignments, we also received feedback from students’ course evaluations on workload within the initial courses developed; we recognized a need to reconsider the concept of contact time. Contact time was not an initial consideration in designing the first courses. However, it soon became a critical factor, as it influences accreditation standards and determines financial aid eligibility for many of our adult learners.

In determining course materials and assignments, we followed the policy that students attending three-credit-hour courses in our programs should expect to spend, on average, 16–20 hours per week on coursework in a 7-week course. We based this range on two factors: (1) the perceived hours of average work per week stated by our students in course surveys; and (2) the minimum approximation of 135 hours for a 3-credit hour course over the course of seven weeks recommended by the Guidance to Institutions and Accrediting Agencies Regarding a Credit Hour as Defined in the Final Regulations Published on 29 October 2010 [40]. This document, published by the US Department of Education, specifies recommended guidelines for determining credit hours. The designation of credit hour is important for educational accreditors and is the method used to determine student status for federal financial aid. The recommended minimum reasonable approximation involved for one credit hour is 45 hours of student work. For example, in order for a course to be considered a 3-credit hour course, and be eligible for financial aid, it is expected that the approximation of the work to be done by a student throughout the duration of that course is around 135 hours (3 times 45 hours). Prior to adopting the IC format, all 15-week courses were 3-credit-hour courses requiring a minimum of nine hours of student work per week.

### Evaluating and revising

The process of course design did not end after initial best practice guidelines were developed. The fields of study for the programs discussed in this article are constantly evolving. Therefore it was critical to set up a process for evaluation and assessment against the key competencies and student program outcomes to decide on required course revisions. In some cases, the content update was minor and simple. For example, an additional instructional video or reading might have been necessary to promote understanding of course content or assignment specifications. However, at other times, courses required more in-depth revisions, such as revising an assessment or reorganizing materials across weekly sessions to scaffold the learning in new ways.

The course evaluation and revision process allowed us to reflect and identify new case studies or opportunities to include new resources and provide additional examples as necessary to support student achievement of fulfilling the course objectives. To standardize the revision process, the faculty generated common standards that rely on gaining information and feedback from multiple sources. Those include, for example, student evaluations, self-evaluations, peer-to-peer teaching reviews, consultation with another faculty, and consultation with the GW Teaching & Learning Center.

In addition to continual course evaluation feedback and thoughtful revisions, we have also been engaged in a continuous quality improvement process to assess the effectiveness of our courses in comparison to the previous 15-week versions. We are in the process of preparing a second manuscript that presents our findings from this research.

## Lessons-learned

Several considerations related to course requirements and delivery had to be discussed and decided upon to ensure each program fulfilled university and program requirements when the initial two programs were revised from 15-week to 7-week duration. With regard to an organizing structure across courses in all programs, we adopted a revised syllabus format to include instructor contact information and updated university policies. Within this syllabus, though not required at the department level at that time, we also added a revised credit hour policy for the 7-week curriculum, based on university accreditation guidance, financial aid requirements, and student feedback on course evaluations related to hours spent completing all course readings and assignments within the 7-week courses. The inclusion of this statement alerted students early in the course as to the amount of time required each week in a 7-week curricula model.

Regarding contact time, after our initial inclusion of a credit hour policy in the syllabi for 7-week courses, our department decided to perform additional confirmatory research and analysis on minimum requirements for student time spent in hours per week to fulfill 3-credit course requirements. We plan to explore further course evaluations and other relevant data as we gain more experience in determining contact time. Yet, our ‘struggle’ with the concept and the requirement to provide student guidance for the 7-week courses, raised our awareness of the ambiguity in guidelines across different course formats and emphasized the need to adopt a more consistent approach to determining contact time across course formats and modalities, particularly since we have students who take courses in multiple formats.

In terms of curriculum development, scaffolding of assignments to demonstrate mastery of course objectives and development of key competencies remains a ‘best practice guideline.’ In general, we assume that students will have less time to reach higher level learning objectives [41] within any one course and over the duration of the program. Hence, we rely upon scaffolding of assignments within a given course and across courses in a curriculum to efficiently promote higher level learning. For example, the two major assignments in the capstone course include a literature review on the student’s topic of interest that supports the final assignment, a Comprehensive Change proposal. In the literature review, the student identifies and describes literature related to a problem of interest. In the final assignment, he or she must analyze the literature and then integrate knowledge across various sources to address a required change within an organization. Another example is in the first CRA course, where students use the discussion board to practice developing evidence-based arguments in preparation for use in a major assignment.

In addition to scaffolding within a given course, we also scaffolded assignments across courses. For example, the final assignment for our healthcare enterprise course requires that students develop strategic priorities and evaluation metrics for a healthcare enterprise. They then use these priorities and metrics to create a change proposal in their capstone course. This type of scaffolding across courses proves essential to ensuring the development and mastery of required key concepts such as strategic planning, leadership, and change management within our intensive programs.

Due to the decrease in available time to complete assignments, we also made several modifications to our approach to assignment design. After receiving feedback on student workload, we agreed to limit the number of major assignments to a maximum of three. Also, we determined that group assignments requiring collaboration were no longer feasible due to time constraints in the intensive courses, even if the collaboration would achieve higher levels of learning. With regard to assignment specifications, student evaluations indicated a need to be much more ‘prescriptive’ in our instructions. For example, the CRA program director found that assignment instructions necessitated more detail and that templates of desired structures, when provided, helped to ensure student success. In general, students in these ICs ask for exemplars of past assignments, which we provide when appropriate.

A final lesson-learned relates to our ability to create a learning community within our ICs. Creating a sense of community within the classroom helps to bridge transactional distance, or psychological and communication space between learners and the instructor, which can occur in an online environment [42]. We adopt the Community of Inquiry model for online learning to foster community in our online courses in order to bridge the transactional distance and promote the type of meaning negotiation requisite for higher levels of learning [43]. Within this model, cognitive presence, faculty presence, and social presence are required to create an online learning community in which students feel comfortable negotiating controversial topics related to healthcare [43]. Cognitive presence involves ‘the exploration, construction, resolution, and confirmation of understanding through collaboration and reflection in a community of inquiry’ [44, p.65]; social presence relates to ‘the ability to project one’s self and establish personal and purposeful relationships’ [44, p.61]; and teaching presence relates to 'design, facilitation, and direct instruction in an online course' [44, p.67]. While we design our assignments to promote demonstration of cognitive presence (e.g., discussion board postings), the decreased time within an IC raised challenges with ensuring the teaching presence and social presence required within an online learning community.

Because ICs involve a compressed timeframe for delivery, we had to consider how to maximize faculty presence and promote faculty-student and student-student interactions required for social presence to emerge. First, faculty are encouraged to begin each course with a Blackboard Collaborate session in which both faculty and students can review the course structure and get to ‘see’ and hear one another in the online classroom. Collaborate is a functionality within Blackboard similar to face-to-face class discussions where sessions can be recorded for those who could not attend. This type of synchronous interaction early in a course helps to establish both the teaching presence and social presence that supports development of cognitive presence as the course progresses and students engage in weekly asynchronous discussions. In addition to initial course introductions, weekly Office Hours, which can be done through Collaborate or other interactive means such as a virtual meeting software like WebEx, support faculty presence within an online course.

Faculty are also encouraged to create videos to either introduce weekly session topics or to present a wrap-up discussion of key learnings at the end of a session to further establish faculty presence. Additionally, in some courses, such as the capstone course, VoiceThread® technology is used for these presentations. VoiceThread® is a cloud based application which allows people to share images, presentations, and videos. Within VoiceThread® viewers can comment on a presentation by typing a message or by recording a voice or video message (www.voicethread.com). Use of this technology by faculty supports teaching presence. In addition, we encourage the use of this technology for student presentations as well because hearing and seeing the video of a presenter and the comments of peers on a presentation helps to develop both the social and cognitive presence required in a learning community.

Finally, we include a Student Lounge/Water Cooler discussion board in most classes. This discussion venue is open only to students and restricts faculty to enter. This discussion board provides a place where students can discuss topics not necessarily related to course content. These types of discussions help to promote social presence and networking for professional development advice within an online course.

## Conclusion

This paper details our iterative process to develop an intensive curriculum from existing traditionally structured courses. Establishing a steering committee that met regularly to discuss the transition processes and formalize lessons-learned as they emerged proved critical to our process of learning and implementation. Even though we initially planned a uniform way of moving forward in program design based upon steering committee interactions, PDs were allowed flexibility to modify their processes to address the needs of a given program. This flexibility allowed PDs to support their faculty designing or redesigning courses to promote achievement of program student outcomes while considering good educational practices and pedagogies. Best practice guidelines continue to emerge as we evaluate the effectiveness of this delivery model and gain more experience over time.

For other programs considering IC models, following our process will help to ensure streamlined courses are aligned to program competencies, without sacrificing essential content. Failing to follow the process could result in misalignment and removal of critical information necessary to ensure achievement of course objectives and corresponding competencies. Also, multi-disciplinary collaboration across programs throughout the process allowed for the development of best-practices and the recognition of lessons-learned from each program for the benefit of all. Failing to ensure this type of collaboration across curricula within a given department/division could inhibit the ability of one program to learn from others as they attempt this type of curricula redesign.

Further research is required regarding the effectiveness of the 7-week IC model compared to the traditional 15-week model of online course delivery with respect to promoting achievement of program student outcomes. Additional research is also required on comparative effectiveness of course facilitation in the two different models of delivery. We are particularly interested in exploring data from student course evaluations and additional direct and indirect measures of learning and student satisfaction between these two models of delivery as a next step in our research. Finally, longitudinal research is required on students participating in ICs within a given program of study and across different programs of study to determine variables influencing success in subsequent courses.
